# Disparities in end-of-life care and place of death in people with malignant brain tumors—A Swedish registry study

**DOI:** 10.1093/nop/npae113

**Published:** 2024-11-11

**Authors:** Anneli Ozanne, Joakim Öhlén, Stina Nyblom, Asgeir Store Jakola, Anja Smits, Cecilia Larsdotter

**Affiliations:** Department of Neurology, Sahlgrenska University Hospital, Gothenburg, Sweden; Institute of Health and Care Sciences, Sahlgrenska Academy, University of Gothenburg, Gothenburg, Sweden; Centre for Person-centred Care (GPCC), Sahlgrenska Academy, University of Gothenburg, Gothenburg, Sweden; Institute of Health and Care Sciences, Sahlgrenska Academy, University of Gothenburg, Gothenburg, Sweden; Institute of Medicine, Sahlgrenska Academy, University of Gothenburg, Gothenburg, Sweden; Palliative Centre, Sahlgrenska University Hospital, Västra Frölunda, Sweden; Department of Neurosurgery, Sahlgrenska University Hospital, Gothenburg, Sweden; Department of Clinical Neuroscience, Institute of Neuroscience and Physiology, Sahlgrenska Academy, University of Gothenburg, Gothenburg, Sweden; Department of Clinical Neuroscience, Institute of Neuroscience and Physiology, Sahlgrenska Academy, University of Gothenburg, Gothenburg, Sweden; Department of Neurology, Sahlgrenska University Hospital, Gothenburg, Sweden; Department of Nursing Science, Sophiahemmet University, Stockholm, Sweden

**Keywords:** brain neoplasms, end-of-life, health services accessibility, palliative care, palliative medicine

## Abstract

**Background:**

Malignant brain tumors often lead to death. While improving future treatments is essential, end-of-life care must also be addressed. To ensure equitable palliative care, understanding the place of death is crucial, as disparities may lead to inequity of care. This study aims to identify the place of death in adults with malignant brain tumors in Sweden, and the potential associations with official palliative care status by the ICD-10 code Z51.5, sociodemographic factors, health service characteristics, and healthcare service utilization.

**Methods:**

A population-level registry study examined the place of death among adults who died of malignant brain tumors in Sweden from 2013 to 2019. Descriptive statistics, univariable, and multivariable binary logistic regression analyses were performed.

**Results:**

We identified 3,888 adults who died from malignant brain tumors. Of these, 64.4% did not receive an official palliative care status. Specialized palliative care was not utilized in 57.2% at the place of death and in 80% of nursing home deaths. In the last month of life, 53.5% of hospital deaths involved 1 transfer, while 41.8% had 2 or more transfers. The odds ratio (OR) of dying in hospital versus at home was higher, with 2 or more transfers (OR 0.63 [0.40, 0.99]). The OR of dying in a hospital versus at home showed significant regional differences.

**Conclusions:**

Despite the severity of their diagnosis, only a minority of patients utilized specialized palliative services at death, and this varied by the place of death. Significant regional disparities were found between hospital and home deaths, indicating unequal end-of-life palliative care in this patient group.

**Key Points**

- Sixty-four percent of adults with malignant brain tumor lacked official palliative care status.
- Fifty-seven percent didn’t use specialized palliative care at death, rising to 80.0% in nursing homes.
- Odds of dying in hospital versus home were higher with ≥2 transfers and varied regionally.

**Importance of the Study**

This registry study of 3,888 adults who died from malignant brain tumors highlights significant disparities in end-of-life palliative care. Over 60% lacked an official palliative care status, and specialized palliative care was not utilized in 57.2% of deaths, rising to 80% in nursing homes. In the last month of life, 53.5% of hospital deaths involved 1 transfer, while 41.8% had 2 or more. The odds of dying in a hospital versus at home were higher with multiple transfers and showed significant regional differences. Despite the severity of their illness, most patients did not receive specialized palliative care, with notable variations by place of death and region. This study underscores the urgent need for equitable palliative care access for all patients.

Place of death is globally recognized as a key policy maker and outcome measure of the quality of end-of-life care,^1,2^ serving as a significant factor in healthcare decision-making. A population-based place of death study from 2012 revealed that 42.1% of all deaths in Sweden occurred in hospitals, 38.1% in nursing homes, and only 17.8% in the person’s own home.^3^ Moreover, from 2013 to 2019, home deaths increased by 1.9%, while hospital deaths decreased by 2.6%.^4^ Despite the perception of one’s own home as an ideal place for death due to its comfort, familiarity, privacy, and safety,^5–7^ hospitals more frequently serve as the place of death.^5,8^

Primary brain tumors and other tumors in the central nervous system (CNS) (malignant and nonmalignant) are the 8th most common cancer in adults over 40 years in the USA.^9,10^ The average annual mortality rate is 4.4/100 000, with an average of 16.2 deaths annually attributed to primary malignant brain and other CNS tumors.^10^ Malignant brain tumors in adults result in a greater loss of potential years of life compared with any other cancer.^11^ The most prevalent type of malignant brain tumor in adults is infiltrative (diffuse) gliomas.^12^ These tumors are incurable at present due to various factors, including their infiltrative nature, the protective role of the blood-brain barrier, and their ability to suppress the immune system.

Cognitive symptoms, focal neurological deficits, and epileptic seizures are common in patients with brain tumors. At progression, symptoms of increased intracranial pressure, such as headache and nausea may appear.^13^ In the last weeks of life, symptoms such as drowsiness, dysphagia, uncontrolled epileptic seizures, headache, death rattle, agitation, and delirium are common.^14^ Quality of life (QoL) and mental health of both patients and their relatives are affected.^15–17^

The healthcare system in Sweden is governed by the Health and Medical Services Act and is characterized by a decentralized structure, with responsibilities shared between regional councils and municipal governments. The regional councils bear the primary responsibility for organizing healthcare services, while municipalities responsible for nursing homes and home care services.^18^ Palliative care seeks to enhance the overall wellbeing of patients and their relatives by addressing and alleviating physical, psychological, social, and existential challenges, thereby preventing and alleviating suffering across all facets of life.^19^ Equal access to care according to needs is a goal in the national Swedish Cancer plan, the national care plan for brain and spinal cord tumors, and the national guidance for palliative care,^20–25^ which among other things, means that there should be no regional differences. Palliative care is integrated into the broader healthcare system and is provided across various settings, including in hospitals, nursing homes, and home care services. Further, palliative care shall be delivered in both specialized and nonspecialized services. General palliative care shall be provided by the healthcare professionals at the respective unit, regardless of the hospital or care facility in which they are located. However, access to specialized palliative care services is known to vary across the country. Hospitals and nursing homes without the availability of specialized palliative care services have the option to consult with specialized palliative consultation teams for support and guidance.^25^

It is essential to ensure the equitable distribution of resources in order to facilitate the provision of equal access to palliative care according to needs, across the country and irrespective of the care setting.^23^ Research indicates that the early integration of specialized palliative care alongside tumor-directed treatments can significantly enhance the quality of care provided to patients.^26^ Despite the severity and progressive symptoms of brain tumors, little research has focused on where these patients die, at home or in healthcare facilities, and the nature of end-of-life care they receive.

In order to improve health policies, distribute resources effectively, and ensure equitable access to palliative care services,^27^ it is crucial to examine the patterns of end-of-life care and the place where people die at a population level. Here, we utilized registry data to identify the place of death in adults with malignant brain tumors in Sweden between 2013 and 2019, and to investigate the potential associations between place of death, official palliative care status by the ICD-10 code Z51.5, sociodemographic factors, health service characteristics and healthcare service utilization at end-of-life.

## Methods

### Design

This population-based study is part of a larger project studying trends in place of death in Sweden.^4^ All the deceased diagnosed with malignant brain tumors at the age of ≥18 between 2013 and 2019 and with a registered place of death were included. The start of the project coincided with the initiation of the first national palliative care policy^23,25^ in Sweden, while 2019 was selected as the study endpoint, based on the assumption that the COVID-19 pandemic may have adversely impacted the place of death. Data was retrieved from the Swedish National Board of Health and Welfare (NBHW), the Swedish Register of Palliative Care (SRCP), and Statistics Sweden (SCB). The NBHW provided death certificate data (sex, age, underlying cause of death, and place of death). The certificate data from each personal identity number of the included participants were linked with patient register data to obtain information regarding hospital transitions during the last month of life. The SRCP provided data on the utilization of specialized palliative care service and SCB provided data on sociodemographic factors.

### Ethical Approval

Since the sample consists of deceased individuals, The Swedish Ethical Review Authority stated that the study could be conducted without ethical vetting (no. 2019-05213, 2020-01758). All data were anonymized and coded by the responsible authority NBHW before being sent to the research group.

### Study Variables

The primary outcome variable was the place of death, categorized into hospital (unspecified specialty; healthcare provided by regions); home (privately owned or rented); nursing home (including short- and long-term residential care settings and other forms of group dwellings); and other (eg, public places, roads, workplace). Healthcare service utilization data included hospital transfers and emergency care during the last month of life, as well as whether specialized palliative services were utilized at the time of death. A dichotomous independent variable, indicating whether specialized palliative care was utilized at the time of death, was formulated based on SRPC data, specifically pertaining to care utilized in a specialized in- or out-patient palliative care service of any nature.

To identify people with potential palliative care needs, a variable based on the Murtagh model^28^ was created. Underlying causes of death from brain tumors were grouped by ICD-code into categories: (a) C71.1 (malignant neoplasm of the frontal lobe); (b) C71.2 (malignant neoplasm of temporal lobe); and (c) C71.0 (malignant neoplasm of cerebrum, except lobes and ventricles), C71.3 (malignant neoplasm of parietal lobe), C71.4 (malignant neoplasm of occipital lobe), C71.6 (malignant neoplasm of cerebellum), C71.7 (malignant neoplasm of brain stem), C71.8 (malignant neoplasm of overlapping sites of brain), and C71.9 (malignant neoplasm of brain, unspecified). This study excludes benign brain tumors, metastatic tumors, and tumors originating from the meningeal sheets that are coded separately. The ICD-10 code Z51.5 was also included to describe the official palliative care status. Z51.5 is a medical classification code used for factors influencing health status and encounters with health services. It is intended to be applied by the physician responsible for the patient’s care when she/he qualifies for palliative care.

Included variables, previously recognized to affect the place of death were sociodemographic characteristics, geographic location, year of death, age at death, healthcare region, and potential palliative care needs.

### Statistical Analyses

Continuous variables were described with mean and SD, and categorical variables with *n* and %. Variations in place of death and its related factors were analyzed by first using univariable binary logistic regression, and then performing multivariable binary logistic regression in the second step. In all these analyses, place of death was used as the dependent variable. To understand patterns in place of death, the analyses were performed separately and stratified according to the living situation of the deceased. Thus, an analysis was performed for those living at home and dying in the hospital versus dying at home. Since only 57 observations were identified for those living in nursing homes, it was not feasible to include them in the multivariable model adaptation.

Three different multivariable models were performed depending on which set of independent variables were used in the model. In model 1, the independent variables included were: sex and age at death. In the second model, all significant individual-related variables from univariable analyses were used. The third model was the same as the second model, but with the addition of healthcare related variables.

The significance tests were 2 sided and conducted at 5% significance level. Goodness of-fit was assessed by using the area under the Receiver Operating Characteristic curve (ROC curve). Statistical analyses were performed using SAS/STAT Software, version 9.4 of the SAS System for Windows (SAS Institute Inc.).

## Results

### Distribution of Place of Death

Between 2013 and 2019, Sweden recorded 3,888 adult fatalities due to malignant brain tumors (41.4% female). Of these, 35% died in hospital, 36% died in a nursing home, and 26.5% died at home. Among all the men, 37% died in hospital, 33.5% died in a nursing home, and 27.1% died at home, while among all the women, 32.2% died in hospital, 39.4% died in a nursing home, and 25.7% died at home. Between 40.2% and 47.6% of those aged 18–59 died in hospital, 11.9–26.1% died in a nursing home, and 30.5–36.9% died at home. Of those aged between 60 and 79, 31.2–35% died in hospital, 35.2–42% died in a nursing home, and 24.3–27.5% died at home (Supplementary Table 1).

Of all the people with malignant brain tumors, 64.4% did not receive the official palliative care status, ICD-10 code Z51.5. Among those who died in a hospital, 56.7% did not receive this palliative care status, while for those who died in a nursing home or died at home, the percentage was 72.7% and 64.8% respectively. Additionally, 57.2% of all people with malignant brain tumors did not utilize specialized palliative care where they died. Among those who died in a nursing home, 80% did not utilize this service. Among all the people with brain tumors who died in hospital, 53.5% had 1 transfer to the hospital 1 month before death, and 41.8% had 2 or more transfers (Supplementary Table 1).

### Regional Variations in Place of Death

**Figure 1.**
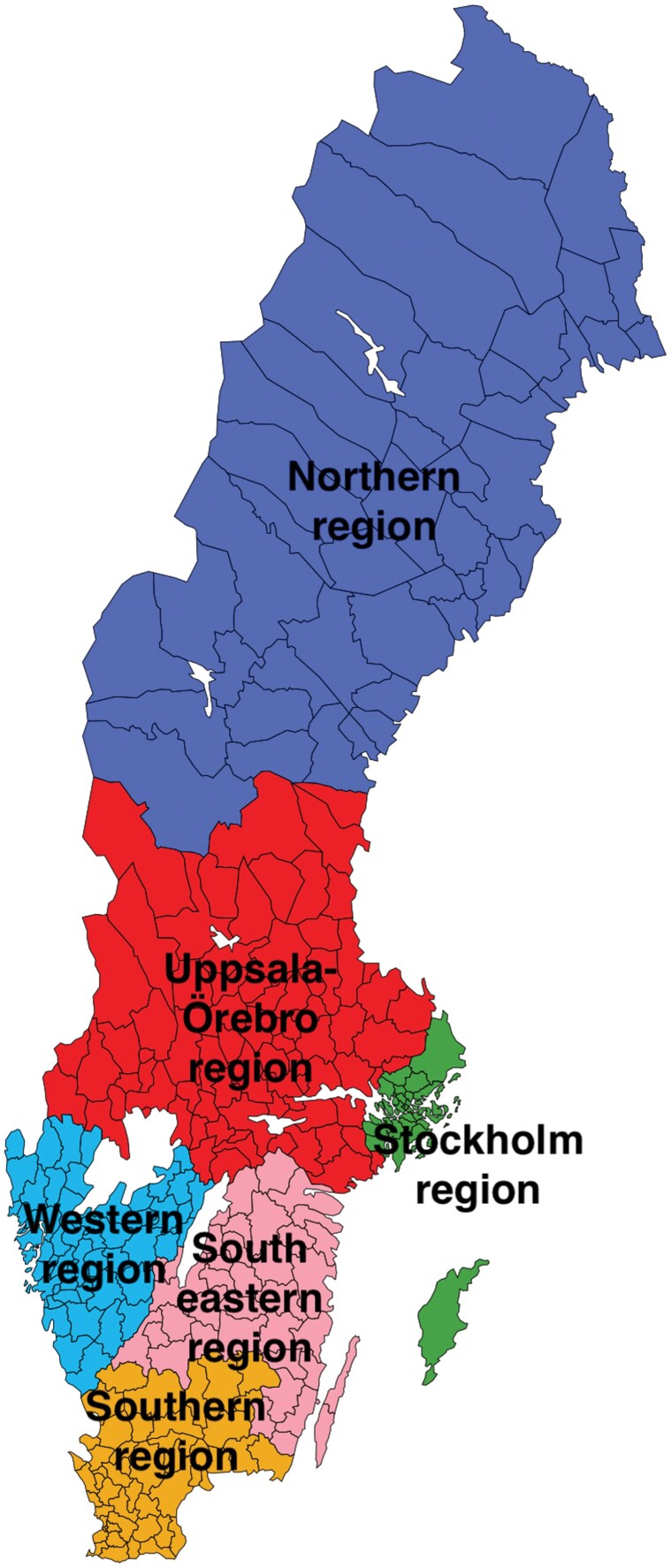
The Swedish regions. License Creative Commons Erkännande-Dela Lika 2.5 Generisk. Webpage: SWE-Map Sjukvårdsregioner-kommuner - Sjukvårdsregion—Wikipedia.

Place of death varied across regions. In the Stockholm region, people with brain tumors most commonly died in hospitals (58.9%), with the lowest percentage of deaths occurring in a nursing home or at home (18.1% respectively). Conversely, the south-eastern region had the lowest proportion of deaths in hospitals (18.5%) and the highest percentage of deaths at home (35.1%), while the western region had the highest percentage of deaths in a nursing home (49.7%) (Supplementary Table 1). Figure 1 shows the regions.

The number of deaths with specialized palliative care services varied across healthcare regions. Of the total population with malignant brain tumors, specialized palliative care services were utilized by only 19.4% of those who passed away in hospital, 7.2% of those who died in nursing homes, and 14.1% of those who died at home. Across regions, the percentage of hospital deaths with specialized palliative care services was highest in Stockholm (48.6%), while the lowest was in the western region (6.2%). For deaths in nursing homes, the highest percentage with specialized palliative care services was in the western region (21.1%), and the lowest in the Uppsala-Örebro region (1.6%). For deaths at home, the lowest percentage with specialized palliative care services was in the western region at 6.3%, compared with 20.3% in the southern region (Table 1).

**Table 1.** Regional Variations in Place of Death, as Related to Healthcare Region and Specialized Palliative Care Services

		Hospital death with specialized palliative care services		Nursing home death with specialized palliative care services		Home death with specialized palliative care services		Death in other place^c^
Malignant brain tumor regardless ICD-10 codes^b^	Healthcare region	No ^a^	Yes ^a^	No ^a^	Yes ^a^	No ^a^	Yes ^a^	
Total	Northern region	**85 (24.1%)**	39 (11.1%)	93 (26.4%)	34 (9.7%)	31 (8.8%)	45 (12.8%)	25 (7.1%)
	Uppsala-Örebro region	162 (19.5%)	139 (16.7%)	294 (35.3%)	*13 (1.6%)*	124 (14.9%)	94 (11.3%)	6 (0.7%)
	Stockholm region	*82 (10.3%)*	**386 (48.6%)**	*112 (14.1%)*	32 (4.0%)	*16 (2.0%)*	128 (16.1%)	39 (4.9%)
	Western region	117 (15.7%)	*46 (6.2%)*	213 (28.6%)	**157 (21.1%)**	**157 (21.1%)**	*47 (6.3%)*	8 (1.1%)
	South-eastern region	52 (11.5%)	32 (7.1%)	**186 (41.1%)**	23 (5.1%)	69 (15.2%)	90 (19.9%)	1 (0.2%)
	Southern region	107 (15.0%)	113 (15.9%)	221 (31.1%)	20 (2.8%)	85 (12.0%)	**144 (20.3%)**	21 (3.0%)
	Total	605 (15.6%)	755 (19.4%)	1119 (28.8%)	279 (7.2%)	482 (12.4%)	548 (14.1%)	100 (2.6%)

Italicized text indicates the lowest percentages of deaths with specialized palliative care services, and bold text indicates the highest.

^a^ *n* (row percentages).

^b^Due to the small number of participants, when divided into ICD-10 codes, the results only show the total number of all malignant brain tumors.

### Multivariable Analyses of Place of Death

In step 1, univariable binomial regression analyses were performed to examine the associations between living at home and dying in the hospital versus dying at home (Supplementary Table 2), with analysis of the variations in place of death and its related factors. The results from the second step, the multivariable binary logistic regression analyses, are described in Model 1 (Supplementary Table 3), Model 2 (Supplementary Table 4), and Model 3 (Table 2).

**Table 2.** Model 3, Multivariable Analyses for Cohort

				Univariable	Multivariable
Variable	*n*	Missing	Hospital (*n* = 1292)	OR (95% CI)	OR (95% CI)
Marital status	2283	0		***P < *.*0001	***P = *.*35
Married			766 (52.6%)		
Unmarried			231 (61.4%)	1.44 (1.14, 1.81) *P* = .002	1.27 (0.87, 1.86) *P* = .21
Widowed			91 (62.8%)	1.52 (1.07, 2.16) *P* = .020	1.54 (0.85, 2.80) *P* = .16
Divorced			204 (66.7%)	1.80 (1.39, 2.33) *P* < .0001	0.99 (0.66, 1.49) *P* = .98
Residing in urban area	2283	0			
No			181 (48.0%)		
Residing in urban area			1111 (58.3%)	1.51 (1.21, 1.89) *P* < .001	1.11 (0.80, 1.55) *P* = .54
Living in single-person household	2283	0			
Single-person household			279 (64.6%)		
Multi-person household			1013 (54.7%)	0.66 (0.53, 0.82) *P* < .001	1.01 (0.68, 1.51) *P* = .96
Palliative care diagnosis ICD-code Z51.5	2283	0			
No			730 (53.1%)		
Yes			562 (61.9%)	1.44 (1.21, 1.70) *P* < .0001	1.05 (0.79, 1.39) *P* = .74
Healthcare Region	2283	0		***P < .0001	***P < .0001
Uppsala-Örebro region			285 (58.0%)		
Northern region			121 (62.1%)	1.18 (0.84, 1.66) *P* = .34	0.85 (0.52, 1.40) *P* = .53
Stockholm region			443 (76.9%)	2.41 (1.85, 3.14) *P* < .0001	1.80 (1.20, 2.71) *P* = .005
Western region			153 (43.5%)	0.56 (0.42, 0.73) *P* < .0001	0.48 (0.32, 0.72) *P* < .001
South-eastern region			78 (33.2%)	0.36 (0.26,0.50) *P* < .0001	0.36 (0.23,0.59) *P* < .0001
Southern region			212 (48.8%)	0.69 (0.53,0.90) *P* = .005	0.55 (0.37,0.80) *P* = .002
Number of hospital transfers	2283	0		***P < .0001	***P < .0001
None			63 (7.9%)		
One transfer			691 (78.2%)	41.94 (30.98, 56.78) *P* < .0001	41.60 (30.16, 57.38) *P* < .0001
Two or more transfers			538 (90.0%)	105.04 (72.50, 152.19) *P* < .0001	114.79 (76.68, 171.84) *P* < .0001
Number of emergency department visits	2283	0		***P < .0001	***P = .13
None			824 (51.4%)		
One unplanned health care visit			345 (69.3%)	2.13 (1.72, 2.64) *P* < .0001	0.88 (0.64, 1.20) *P* = .41
Two or more unplanned health care visits			123 (67.2%)	1.94 (1.40, 2.68) *P* < .0001	0.63 (0.40, 0.99) *P* = .044
Place of death within a specialized palliative care facility	2283	0		***P = .21	***P < .001
No			571 (55.2%)		
Yes			721 (57.8%)	1.11 (0.94, 1.31) *P* = .21	0.62 (0.47, 0.82) *P* < .001

OR is the ratio for the odds of an increase in the predictor of one unit.Area under ROC curve with 95% CI for multivariable model = 0.90 (0.89, 0.91).

Abbreviations: CI, confidence interval; IQR, interquartile range; SD, standard deviation; OR, odds ratio.

***P-value for the entire effect/factor/variable.

**Figure 2.**
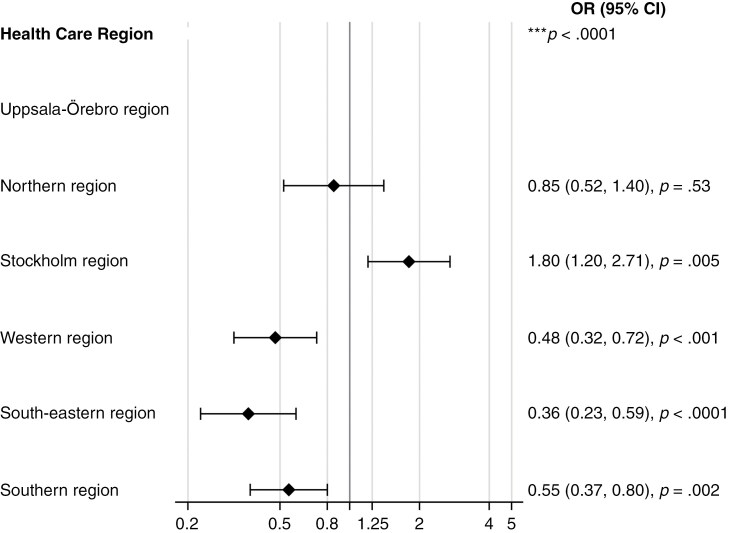
Multivariable analyses for people residing at home and dying in a hospital versus at home (with health region and adjusted for significant variables).

The multivariable analysis showed that the OR of dying in hospital versus at home varied according to the healthcare region where the person resided. For example, for people living at home in the Stockholm region, there was a higher OR of dying in the hospital compared with dying at home (1.20, 2.71; *P* = .005). In contrast, the OR of dying at home compared with dying in the hospital was higher across the western region (0.32, 0.72; *P* < .001), south-eastern region (0.23, 0.59; *P* < .0001), and the southern region (0.37, 0.80; *P* = .002). Additionally, among those who resided at home, the OR of dying in the hospital versus at home was higher for people who had 1 (30.16, 57.38; *P* < .0001) or more (76.68, 171.84; *P* < .0001) transfers to the hospital in the last month of life. The OR of dying in hospital versus at home was lower for people who utilized specialized palliative care services at death (0.47, 0.82; *P* < .001) (Table 2; Figure 2).

## Discussion

This registry study of 3,888 adults with malignant brain tumors highlights significant disparities in the end-of-life situation across different regions of the country. We found regional disparities in the place of death, with hospital deaths overrepresented in the capital region, Stockholm. In the western, south-eastern, and southern regions, the odds of dying at home were significantly higher.

In our study, only a minority of all adults with malignant brain tumors utilized specialized palliative care services at death despite their severe diagnosis. All patients with malignant brain tumors were included; however, without the ability to stratify them by severity, it is possible that the utilization of specialized palliative care services was greater among patients with high-grade tumors compared with those with low-grade tumors. Bearing in mind that the place of death is a key policy maker for the quality of end-of-life care,^1,2^ these findings raise concerns regarding the pursuit of equitable care. Despite the existence of both European and national guidelines in neuro-oncology and palliative care,^24,29,30^ there appear to be deficiencies in the routines that facilitate the utilization of specialized palliative care services. While patients are expected to have access to general palliative care across all care facilities, the availability of specialized palliative care services varies. In settings and situations where specialized palliative care services are not directly accessible, consultation with specialized palliative care providers could have been provided.

In our study, we lack information on the circumstances influencing the location of death across different regions. Possibly, the variations observed may depend on infrastructural or organizational factors, such as differences in availability and types of specialized palliative care services across different regions.^31^ While previous research highlights the roles of geographic, socioeconomic factors, and individual factors in determining the place of death,^3,32^ these factors are insufficient to explain the variation in our study. Gao et al.^33^ propose that the influence of health service infrastructure and organizational factors on the place of death requires further investigation. This calls for a focused examination of palliative care policies, particularly in relation to types of care services, levels of palliative care, service capacity, and geographical coverage, which we have studied in a yet unpublished study. Sweden’s bifurcated system of responsibility and organization, wherein municipalities manage nursing homes and regions oversee hospitals, likely complicates the effective integration of palliative care. National Board of Health and Welfare in Sweden has also reported significant regional disparities in the provision of palliative care, which may be linked to variations in staff training, symptom identification and management, end-of-life discussions, care planning, and support for family carers.^34^

The fact that 41.8%–53.5% of those who died in hospital had 1 or more transfers 1 month before death might be due to multiple factors, such as the severity of the disease, its complications and symptoms, and/or a lack of an advanced care plan. It is alarming that almost 2 out of 3 patients with malignant brain tumors lacked official palliative care status by the ICD-10 code Z51.5. However, this does not appear to be related to the place of death. Symptoms such as cognitive effects, neurological deficits, uncontrolled seizures, and progressive headaches, among others^13^ might be difficult to manage at home. The clinical impression is that some patients have the perception that hospital care provides a safer environment for families caring for a loved one during end-of-life care at home. Possibly, such factors will have influenced the place of death and the transfers to the hospital.

This study and others^35–37^ have found transfers to a hospital in the last months of life to be common. Additionally, other studies have found that comorbidities, higher age, and the malignancy grade of the brain tumor are associated with a greater burden of care^35^ and neurological decline might increase hospitalization in the last month of life.^36^ This likely led to the high number of people dying in hospitals in our study. However, it does not explain the differences in the place of death between regions. Other circumstances, and probably organizational health care issues, may affect where people with malignant brain tumors die.

A Swedish registry study^37^ investigating acute healthcare utilization in the capital region revealed an increase in the use of specialized palliative care services during the last year of life and found that 77% of the patients utilized specialized palliative care within the last 3 months of life. These results contrast with our national results, which showed that only 42.8% utilized specialized palliative care during the last month of life. However, patients receiving specialized palliative care had fewer unplanned emergency room visits and hospitalizations in the last month of life.^38^ This study and others have found that more people without specialized palliative care died in hospital compared with those who received it,^37^ which should argue for the implementation of early integration of specialized palliative care in patients with brain tumors.

Equal care is 1 of the main goals of the Swedish Cancer plan,^24^ implying that referrals to specialized palliative care should not depend on factors such as sociodemographics or comorbidity. Regardless of life circumstances, specialized palliative care must be offered when necessary.^22,39,40^ Having established that inequitable EOL care actually exists, further research into the reasons behind this should be encouraged to potentially correct such an imbalance in the future.

### Strengths and Limitations

One strength of this study is the population-based design with national registers. However, death certificate data (NBHW registry) do not specify whether the place of death was within specialized palliative care services. Utilization of specialized palliative care services at death was determined from data from the SRPC. In 2019, this register had a coverage rate of 60% of all deaths in Sweden, but it is known to cover close to all deaths in specialized palliative care services. The SRPC was thus used in coordination with death registers from the NBHW for calculations of the proportion of the population included in this study that utilized specialized palliative care services. The SRCP exclusively addresses aspects related to the final week of life.

Further limitations are that the degree of malignancy, as well as comorbidities, were not studied, and that some factors already known to influence the place of death were not included, such as the patient’s preferred place of death, living in a socio-economically deprived area, functional status, intensity of home care use, ethnicity, family carer support, integration of home and hospital care services, and to what extent the services had multidisciplinary teams.^41^

## Supplementary Material

Supplementary material is available online at *Neuro-Oncology Practice* (https://academic.oup.com/nop/).

npae113_suppl_Supplementary_Materials

## Data Availability

The data from this study is available from each register holder. Certain restrictions may apply. Programming codes are available from the authors upon reasonable request.
