# Supplementary material for: Disparities in end-of-life care and place of death in people with malignant brain tumors—A Swedish registry study
Source: Neurooncol Pract. 2024 Nov 11;12(3):511–9. doi: 10.1093/nop/npae113 (PMC12137219; doi:10.1093/nop/npae113)
Supplement: npae113_suppl_Supplementary_Materials [file npae113_suppl_supplementary_materials.doc]

**Supplementary Table 1****.** Distribution of place of death of adult population with malignant brain tumor in Sweden from 2013 to 2019.

| **Variable** | **% of total malignant brain tumor deaths** | **Total deaths (n=3888)** | **Hospital deaths (n=1360, 35.0%) ^a^** | **Nursing home deaths (n=1398, 36.0%)** | **Home deaths (n=1030, 26.5%)** | **Other place or unknown place of death (n=100, 2.6%)** |  |
| --- | --- | --- | --- | --- | --- | --- | --- |
| **Sex** |  |  | | | | |  |
| Male | 58.6% | 2277 (100.0%) | 842 (37.0%) | 763 (33.5%) | 616 (27.1%) | 56 (2.5%) |  |
| Female | 41.4% | 1611 (100.0%) | 518 (32.2%) | 635 (39.4%) | 414 (25.7%) | 44 (2.7%) |  |
| **Age at death** |  |  | | | | |  |
| Age18-29 | 2.1% | 84 (100.0%) | 40 (47.6%) | 10 (11.9%) | 31 (36.9%) | 3 (3.6%) |  |
| Age30-39 | 3.6% | 139 (100.0%) | 62 (44.6%) | 25 (18.0%) | 48 (34.5%) | 4 (2.9%) |  |
| Age40-49 | 8.4% | 325 (100.0%) | 134 (41.2%) | 81 (24.9%) | 99 (30.5%) | 11 (3.4%) |  |
| Age50-59 | 16.8% | 652 (100.0%) | 262 (40.2%) | 170 (26.1%) | 202 (31.0%) | 18 (2.8%) |  |
| Age60-69 | 27.4% | 1066 (100.0%) | 373 (35.0%) | 375 (35.2%) | 293 (27.5%) | 25 (2.3%) |  |
| Age70-79 | 28.2% | 1097 (100.0%) | 342 (31.2%) | 461 (42.0%) | 267 (24.3%) | 27 (2.5%) |  |
| Age80-89 | 11.9% | 464 (100.0%) | 132 (28.4%) | 238 (51.3%) | 83 (17.9%) | 11 (2.4%) |  |
| Age90+ | 1.6% | 61 (100.0%) | 15 (24.6%) | 38 (62.3%) | 7 (11.5%) | 1 (1.6%) |  |
| **Localization of malignant brain tumor ICD-10 codes** |  |  | | | | |  |
| Malignant neoplasm in frontal lobe (C71.1) | 3.8% | 149 (100.0%) | 49 (32.9%) | 56 (37.6%) | 41 (27.5%) | 3 (2.0%) |  |
| Malignant neoplasm in temporal lobe (C71.2) | 3.0% | 115 (100.0%) | 39 (33.9%) | 52 (45.2%) | 21 (18.3%) | 3 (2.6%) |  |
| Other localization of the brain tumor (C71.0, C71.3-C71.9) | 93.2% | 3624 (100.0%) | 1272 (35.1%) | 1290 (35.6%) | 968 (26.7%) | 94 (2.6%) |  |
| **Official palliative care status (Z51.5)** |  |  | | | | |  |
| No | 64.4% | 2504 (100.0%) | 771 (30.8%) | 1017 (40.6%) | 667 (26.6%) | 49 (2.0%) |  |
| Yes | 35.6% | 1384 (100.0%) | 589 (42.6%) | 381 (27.5%) | 363 (26.2%) | 51 (3.7%) |  |
| **Living situation ^b^** |  |  | | | | |  |
| Home | 94.0% | 3654 (100.0%) | 1292 (35.4%) | 1273 (34.8%) | 991 (27.1%) | 98 (2.7%) |  |
| Owned residence |  | 2826 (100.0%) | 990 (35.0%) | 954 (33.8%) | 799 (28.3%) | 83 (2.9%) |  |
| Rented residence |  | 828 (100.0%) | 302 (36.5%) | 319 (38.5%) | 192 (23.2%) | 15 (1.8%) |  |
| Nursing home | 1.9% | 73 (100.0%) | 14 (19.2%) | 54 (74.0%) | 5 (6.8%) | 0 (0.0%) |  |
| Other | 1.5% | 57 (100.0%) | 20 (35.1%) | 22 (38.6%) | 15 (26.3%) | 0 (0.0%) |  |
| **Household situation ^b^** |  |  | | | | |  |
| Single-person household | 25.7% | 998 (100.0%) | 309 (31.0%) | 497 (49.8%) | 169 (16.9%) | 23 (2.3%) |  |
| Multi-person household | 73.8% | 2869 (100.0%) | 1044 (36.4%) | 892 (31.1%) | 857 (29.9%) | 76 (2.6%) |  |
| Children under 18 in the household | 11.9% | 462 (100.0%) | 201 (43.5%) | 118 (25.5%) | 133 (28.8%) | 10 (2.2%) |  |
| **Marital status ^b^** |  |  | | | | |  |
| Married | 56.9% | 2213 (100.0%) | 789 (35.7%) | 656 (29.6%) | 708 (32.0%) | 60 (2.7%) |  |
| Unmarried | 17.3% | 673 (100.0%) | 252 (37.4%) | 242 (36.0%) | 157 (23.3%) | 22 (3.3%) |  |
| Widow | 10.0% | 387 (100.0%) | 100 (25.8%) | 223 (57.6%) | 57 (14.7%) | 7 (1.8%) |  |
| Divorced | 15.8% | 615 (100.0%) | 219 (35.6%) | 277 (45.0%) | 108 (17.6%) | 11 (1.8%) |  |
| **Year of Death** |  |  | | | | |  |
| 2013 | 12.1% | 471 (100.0%) | 165 (35.0%) | 178 (37.8%) | 124 (26.3%) | 4 (0.8%) |  |
| 2014 | 12.1% | 469 (100.0%) | 171 (36.5%) | 164 (35.0%) | 128 (27.3%) | 6 (1.3%) |  |
| 2015 | 13.8% | 537 (100.0%) | 179 (33.3%) | 188 (35.0%) | 161 (30.0%) | 9 (1.7%) |  |
| 2016 | 14.4% | 559 (100.0%) | 206 (36.9%) | 201 (36.0%) | 138 (24.7%) | 14 (2.5%) |  |
| 2017 | 16.7% | 651 (100.0%) | 230 (35.3%) | 236 (36.3%) | 166 (25.5%) | 19 (2.9%) |  |
| 2018 | 15.6% | 605 (100.0%) | 201 (33.2%) | 221 (36.5%) | 161 (26.6%) | 22 (3.6%) |  |
| 2019 | 15.3% | 596 (100.0%) | 208 (34.9%) | 210 (35.2%) | 152 (25.5%) | 26 (4.4%) |  |
| **Educational attainment ^b^** |  |  | | | | |  |
| No formal or elementary education | 16.2% | 628 (100.0%) | 192 (30.6%) | 283 (45.1%) | 139 (22.1%) | 14 (2.2%) |  |
| Lower secondary education | 10.6% | 411 (100.0%) | 130 (31.6%) | 156 (38.0%) | 115 (28.0%) | 10 (2.4%) |  |
| Higher secondary education | 47.1% | 1830 (100.0%) | 633 (34.6%) | 648 (35.4%) | 499 (27.3%) | 50 (2.7%) |  |
| Higher education | 25.2% | 978 (100.0%) | 389 (39.8%) | 301 (30.8%) | 262 (26.8%) | 26 (2.7%) |  |
| **Residing in urban area ^b^** |  |  | | | | |  |
| No | 15.5% | 604 (100.0%) | 185 (30.6%) | 205 (33.9%) | 202 (33.4%) | 12 (2.0%) |  |
| Yes | 84.5% | 3284 (100.0%) | 1175 (35.8%) | 1193 (36.3%) | 828 (25.2%) | 88 (2.7%) |  |
| **Healthcare Region ^b^** |  |  | | | | |  |
| Uppsala-Örebro region | 21.4% | 832 (100.0%) | 301 (36.2%) | 307 (36.9%) | 218 (26.2%) | 6 (0.7%) |  |
| Northern region | 9.1% | 352 (100.0%) | 124 (35.2%) | 127 (36.1%) | 76 (21.6%) | 25 (7.1%) |  |
| Stockholm region | 20.4% | 795 (100.0%) | 468 (58.9%) | 144 (18.1%) | 144 (18.1%) | 39 (4.9%) |  |
| Western region | 19.2% | 745 (100.0%) | 163 (21.9%) | 370 (49.7%) | 204 (27.4%) | 8 (1.1%) |  |
| South-eastern region | 11.7% | 453 (100.0%) | 84 (18.5%) | 209 (46.1%) | 159 (35.1%) | 1 (0.2%) |  |
| Southern region | 18.2% | 711 (100.0%) | 220 (30.9%) | 241 (33.9%) | 229 (32.2%) | 21 (3.0%) |  |
| **Country of birth** |  |  | | | | |  |
| Born in Sweden | 87.5% | 3403 (100.0%) | 1157 (34.0%) | 1267 (37.2%) | 896 (26.3%) | 83 (2.4%) |  |
| Born outside Sweden | 12.5% | 485 (100.0%) | 203 (41.9%) | 131 (27.0%) | 134 (27.6%) | 17 (3.5%) |  |
| **Potential palliative care needs** |  |  | | | | |  |
| Yes | 100% | 3888 (100.0%) | 1360 (35.0%) | 1398 (36.0%) | 1030 (26.5%) | 100 (2.6%) |  |
| **Utilizing palliative care service at death ^c^** |  |  | | | | |  |
| No | 57.2% | 2224 (100.0%) | 605 (27.2%) | 1119 (50.3%) | 482 (21.7%) | 18 (0.8%) |  |
| Yes | 42.8% | 1664 (100.0%) | 755 (45.4%) | 279 (16.8%) | 548 (32.9%) | 82 (4.9%) |  |
| **Number of hospital transfers in last month of life** |  |  | | | | |  |
| None | 46.1% | 1793 (100.0%) | 65 (3.6%) | 934 (52.1%) | 768 (42.8%) | 26 (1.5%) |  |
| One transfer | 34.0% | 1323 (100.0%) | 727 (55.0%) | 356 (26.9%) | 197 (14.9%) | 43 (3.3%) |  |
| Two or more transfers | 19.9% | 772 (100.0%) | 568 (73.6%) | 108 (14.0%) | 65 (8.4%) | 31 (4.0%) |  |
| **Number of ED* visits last month in life** |  |  | | | | |  |
| None | 74.3% | 2889 (100.0%) | 855 (29.6%) | 1145 (39.6%) | 807 (27.9%) | 82 (2.8%) |  |
| One unplanned healthcare visit | 18.8% | 729 (100.0%) | 378 (51.9%) | 178 (24.4%) | 160 (21.9%) | 13 (1.8%) |  |
| Two or more unplanned health care visits | 6.9% | 270 (100.0%) | 127 (47.0%) | 75 (27.8%) | 63 (23.3%) | 5 (1.9%) |  |
| **^a^** For categorical variables n (row %) is presented.  **^b^** Inpatient specialized palliative care or hospice services, or specialized palliative home care services.  **^c^** Independent specialized palliative or hospice services, or specialized palliative home care services  *Emergency department | | | | | | |  |

**Supplementary Table 2** Univariable binomial regression analyses (living at home and

dying in hospital vs. dying at home) all associated variables.

| Variable | n | n missing | Value | n (%) of event | OR (95%CI) Place of Death | p-value | Area under ROC-Curve (95%CI) |
| --- | --- | --- | --- | --- | --- | --- | --- |
| Year of Death | 2283 | 0 | 2013 vs 2013 | 155 (56.6%) | 1.00 | 0.69*** |  |
|  |  |  | 2014 vs 2013 | 164 (57.3%) | 1.03 (0.74-1.44) | 0.85 |  |
|  |  |  | 2015 vs 2013 | 167 (52.2%) | 0.84 (0.61-1.16) | 0.29 |  |
|  |  |  | 2016 vs 2013 | 195 (59.3%) | 1.12 (0.81-1.55) | 0.50 |  |
|  |  |  | 2017 vs 2013 | 218 (57.4%) | 1.03 (0.75-1.41) | 0.84 |  |
|  |  |  | 2018 vs 2013 | 192 (55.7%) | 0.96 (0.70-1.33) | 0.82 |  |
|  |  |  | 2019 vs 2013 | 201 (57.6%) | 1.04 (0.76-1.44) | 0.80 | 0.52 (0.50-0.55) |
| Year of Death continuous | 2283 | 0 | 2013-<2016 | 486 (55.2%) |  |  |  |
|  |  |  | 2016-<2018 | 413 (58.3%) |  |  |  |
|  |  |  | 2018-2019 | 393 (56.6%) | 1.01 (0.97-1.05) | 0.72 | 0.50 (0.48-0.53) |
| Sex | 2283 | 0 | Male | 798 (57.5%) |  |  |  |
|  |  |  | Female | 494 (55.3%) | 0.91 (0.77-1.08) | 0.30 | 0.51 (0.49-0.53) |
| Age at death | 2283 | 0 | Age 60-69 (reference) vs Age 60-69 (reference) | 356 (55.6%) | 1.00 | 0.93*** |  |
|  |  |  | Age 18-29 vs Age 60-69 (reference) | 38 (56.7%) | 1.05 (0.63-1.74) | 0.86 |  |
|  |  |  | Age 30-39 vs Age 60-69 (reference) | 57 (55.9%) | 1.01 (0.66-1.54) | 0.96 |  |
|  |  |  | Age 40-49 vs Age 60-69 (reference) | 123 (56.7%) | 1.04 (0.77-1.42) | 0.79 |  |
|  |  |  | Age 50-59 vs Age 60-69 (reference) | 252 (56.6%) | 1.04 (0.82-1.33) | 0.74 |  |
|  |  |  | Age 70-79 vs Age 60-69 (reference) | 331 (55.9%) | 1.01 (0.81-1.27) | 0.92 |  |
|  |  |  | Age 80-89 vs Age 60-69 (reference) | 124 (61.1%) | 1.25 (0.91-1.73) | 0.17 |  |
|  |  |  | Age 90+ vs Age 60-69 (reference) | 11 (64.7%) | 1.46 (0.53-4.00) | 0.46 | 0.51 (0.49-0.54) |
| Living conditions, Number of children under 18 years | 2283 | 0 | No children under 18 years | 1098 (56.1%) |  |  |  |
|  |  |  | Children under 18 years | 194 (59.5%) | 1.15 (0.91-1.46) | 0.25 | 0.51 (0.49-0.52) |
| Marital status | 2283 | 0 | Married vs Married | 766 (52.6%) | 1.00 | <0.0001*** |  |
|  |  |  | Unmarried vs Married | 231 (61.4%) | 1.44 (1.14-1.81) | 0.0023 |  |
|  |  |  | Widowed vs Married | 91 (62.8%) | 1.52 (1.07-2.16) | 0.020 |  |
|  |  |  | Divorced vs Married | 204 (66.7%) | 1.80 (1.39-2.33) | <0.0001 | 0.56 (0.53-0.58) |
| Educational attainment | 2257 | 26 | Higher secondary education vs Higher secondary education | 612 (56.1%) | 1.00 | 0.41*** |  |
|  |  |  | No formal or elementary education vs Higher secondary education | 178 (56.9%) | 1.03 (0.80-1.33) | 0.82 |  |
|  |  |  | Lower secondary education vs Higher secondary education | 122 (52.6%) | 0.87 (0.65-1.15) | 0.32 |  |
|  |  |  | Higher education vs Higher secondary education | 366 (58.8%) | 1.12 (0.91-1.36) | 0.28 | 0.52 (0.50-0.54) |
| Residing in urban area | 2283 | 0 | NO | 181 (48.0%) |  |  |  |
|  |  |  | Residing in urban area | 1111 (58.3%) | 1.51 (1.21-1.89) | 0.0002 | 0.53 (0.51-0.54) |
| Country of birth | 2283 | 0 | Born in Sweden | 1097 (56.0%) |  |  |  |
|  |  |  | Born outside Sweden | 195 (60.4%) | 1.20 (0.94-1.52) | 0.14 | 0.51 (0.50-0.53) |
| Living in single-person household | 2283 | 0 | Single-person household | 279 (64.6%) |  |  |  |
|  |  |  | Multi-person household | 1013 (54.7%) | 0.66 (0.53-0.82) | 0.0002 | 0.53 (0.51-0.55) |
| Number of emergency department visits | 2283 | 0 | None vs None | 824 (51.4%) | 1.00 | <0.0001*** |  |
|  |  |  | One unplanned health care visit vs None | 345 (69.3%) | 2.13 (1.72-2.64) | <0.0001 |  |
|  |  |  | Two or more unplanned health care visits vs None | 123 (67.2%) | 1.94 (1.40-2.68) | <0.0001 | 0.57 (0.56-0.59) |
| Health Care Region | 2283 | 0 | Uppsala-Örebro region (ref) vs Uppsala-Örebro region(ref) | 285 (58.0%) | 1.00 | <0.0001*** |  |
|  |  |  | Northern region vs Uppsala-Örebro region (ref) | 121 (62.1%) | 1.18 (0.84-1.66) | 0.34 |  |
|  |  |  | Stockholm region vs Uppsala-Örebro region (ref) | 443 (76.9%) | 2.41 (1.85-3.13) | <0.0001 |  |
|  |  |  | Western region vs Uppsala-Örebro region (ref) | 153 (43.5%) | 0.56 (0.42-0.73) | <0.0001 |  |
|  |  |  | South-eastern region vs Uppsala-Örebro region (ref) | 78 (33.2%) | 0.36 (0.26-0.50) | <0.0001 |  |
|  |  |  | Southern region vs Uppsala-Örebro region (ref) | 212 (48.8%) | 0.69 (0.53-0.90) | 0.0052 | 0.66 (0.64-0.68) |
| Palliative care diagnosis ICD-code Z51.5 | 2283 | 0 | No | 730 (53.1%) |  |  |  |
|  |  |  | Yes | 562 (61.9%) | 1.44 (1.21-1.70) | <0.0001 | 0.54 (0.52-0.56) |
| Place of death within a specialized palliative care facility | 2283 | 0 | No | 571 (55.2%) |  |  |  |
|  |  |  | Yes | 721 (57.8%) | 1.11 (0.94-1.31) | 0.21 | 0.51 (0.49-0.53) |
| Potential palliative care needs | 2283 | 0 | Potential palliative care needs | 1292 (56.6%) | . (.-.) | . | 0.50 (0.50-0.50) |
| Localization of malignant brain tumor ICD-10 codes | 2283 | 0 | Malignant neoplasm in frontal lobe (C71.1) vs Malignant neoplasm in frontal lobe (C71.1) | 45 (54.2%) | 1.00 | 0.31*** |  |
|  |  |  | Malignant neoplasm in temporal lobe (C71.2) vs Malignant neoplasm in frontal lobe (C7.1) | 39 (66.1%) | 1.65 (0.83-3.29) | 0.16 |  |
|  |  |  | Other localization of the brain tumor (C71.0, C71.3-C71.9) vs Malignant neoplasm in frontal lobe (C7 | 1208 (56.4%) | 1.09 (0.70-1.70) | 0.69 | 0.51 (0.50-0.52) |
| Number of hospital transfers | 2283 | 0 | None vs None | 63 (7.9%) | 1.00 | <0.0001*** |  |
|  |  |  | One transfer vs None | 691 (78.2%) | 41.94 (30.98-56.77) | <0.0001 |  |
|  |  |  | Two or more transfers vs None | 538 (90.0%) | 105.03 (72.51-152.15) | <0.0001 | 0.87 (0.86-0.89) |
| All tests are performed with univariable logistic regression P-values, OR and Area under ROC-curve are based on original values and not on stratified groups OR is the ratio for the odds of an increase in the predictor of one unit ***) p-value for the entire effect/factor/variable | | | | | | | |

**Supplementary Table 3.** Model 1, multivariable analyses for people residing at home and dying in hospital vs at home.

|  | | | | Univariable | Multivariable |
| --- | --- | --- | --- | --- | --- |
| Variable | missing | n | Hospital (n=1292) | OR (95% CI) | OR (95% CI) |
| Sex | 0 | 2283 |  |  |  |
| Male |  |  | 798 (57.5%) |  |  |
| Female |  |  | 494 (55.3%) | 0.91(0.77,1.08) p=0.30 | 0.91(0.77,1.08) p=0.29 |
| Age at death | 0 | 2283 |  | ***p=0.93 | ***p=0.92 |
| Age 60-69 |  |  | 356 (55.6%) |  |  |
| Age 18-29 |  |  | 38 (56.7%) | 1.05(0.63,1.74) p=0.86 | 1.05(0.63,1.75) p=0.85 |
| Age 30-39 |  |  | 57 (55.9%) | 1.01(0.66,1.54) p=0.96 | 1.01(0.66,1.54) p=0.96 |
| Age 40-49 |  |  | 123 (56.7%) | 1.04(0.77,1.42) p=0.79 | 1.04(0.76,1.42) p=0.81 |
| Age 50-59 |  |  | 252 (56.6%) | 1.04(0.82,1.33) p=0.74 | 1.04(0.82,1.33) p=0.73 |
| Age 70-79 |  |  | 331 (55.9%) | 1.01(0.81,1.27) p=0.92 | 1.01(0.81,1.27) p=0.92 |
| Age 80-89 |  |  | 124 (61.1%) | 1.25(0.91,1.73) p=0.17 | 1.26(0.91,1.74) p=0.17 |
| Age 90+ |  |  | 11 (64.7%) | 1.46(0.53,4.01) p=0.46 | 1.47(0.54,4.02) p=0.46 |
| OR is the ratio for the odds of an increase in the predictor of one unit Area under ROC-curve with 95% CI for multivariable model =0.53(0.50,0.55) ***) p-value for the entire effect/factor/variable Abbreviations: CI, confidence interval; SD, standard deviation, IQR, interquartile range | | | | | |

**Supplementary Table 4 Model 2, multivariable analyses for people residing at home and dying in hospital vs at home.**

|  | | | | Univariable | Multivariable |
| --- | --- | --- | --- | --- | --- |
| Variable | n | missing | Hospital (n=1292) | OR (95% CI) | OR (95% CI) |
| Marital status | 2283 | 0 |  | ***p<.0001 | ***p=0.002 |
| Married |  |  | 766 (52.6%) |  |  |
| Unmarried |  |  | 231 (61.4%) | 1.44(1.14,1.81) p=0.002 | 1.39(1.08,1.79) p=0.011 |
| Widowed |  |  | 91 (62.8%) | 1.52(1.07,2.16) p=0.020 | 1.44(0.96,2.15) p=0.079 |
| Divorced |  |  | 204 (66.7%) | 1.80(1.39,2.33) p<.0001 | 1.70(1.27,2.27) p<.001 |
| Residing in urban area | 2283 | 0 |  |  |  |
| NO |  |  | 181 (48.0%) |  |  |
| Residing in urban area |  |  | 1111 (58.3%) | 1.51(1.21,1.89) p<.001 | 1.45(1.16,1.82) p=0.001 |
| Living in single-person household | 2283 | 0 |  |  |  |
| Single-person household |  |  | 279 (64.6%) |  |  |
| Multi-person household |  |  | 1013 (54.7%) | 0.66(0.53,0.82) p<.001 | 0.91(0.69,1.20) p=0.52 |
| Palliative care diagnosis ICD-code Z51.5 | 2283 | 0 |  |  |  |
| No |  |  | 730 (53.1%) |  |  |
| Yes |  |  | 562 (61.9%) | 1.44(1.21,1.70) p<.0001 | 1.44(1.21,1.71) p<.0001 |
| OR is the ratio for the odds of an increase in the predictor of one unit Area under ROC-curve with 95% CI for multivariable model =0.59(0.57,0.61) ***) p-value for the entire effect/factor/variable Abbreviations: CI, confidence interval; SD, standard deviation, IQR, interquartile range | | | | | |
